# Redefining Reconstruction: Technological Innovations in Microsurgical Breast Reconstruction

**DOI:** 10.3390/cancers17233739

**Published:** 2025-11-22

**Authors:** Nicole E. Speck, Jian Farhadi

**Affiliations:** 1 Department of Plastic Surgery, University Hospital Basel, 4031 Basel, Switzerland; specknicole.e@gmail.com; 2 Plastic Surgery Group, 8008 Zurich, Switzerland; 3 Faculty of Medicine, University of Basel, 4031 Basel, Switzerland

**Keywords:** microsurgery, mammoplasty, robotic surgical procedures, augmented reality, artificial intelligence, anastomosis, surgical

## Abstract

**Background**: Microsurgical breast reconstruction is advancing rapidly with the integration of innovative technologies that enhance surgical precision, safety, and outcomes. This narrative review highlights recent developments across four key phases: flap planning, flap harvest, microvascular anastomosis, and flap monitoring. **Methods**: To identify the most updated and relevant data, all content on «Aesthetic and Reconstructive Breast Surgery Network» (ARBS Network, Copyright 2025 Mark Allen Group, United Kingdom) was screened regarding new technology. The contributions were grouped into one of four key phases. More references related to the content viewed were then searched on the electronic database MEDLINE (Bethesda, MD: U.S. National Library of Medicine). **Results**: 24 contributions regarding new technology were identified on ARBS Network. Of these, 17 were relevant for this paper. Preoperative tools such as CT angiography and AI-based perforator mapping optimize surgical planning and execution. Robotic-assisted or endoscopic techniques for deep inferior epigastric perforator (DIEP) flap harvest enable minimally invasive dissection with reduced donor-site morbidity and improved muscle preservation. Robotic microsurgery, particularly with the MUSA and Symani^®^ Surgical System, allows for precise, tremor-free suturing of submillimeter vessels. Indocyanine green (ICG) angiography remains the gold standard for intraoperative perfusion evaluation. Postoperative flap surveillance is crucial for detecting vascular compromise early. Devices such as the Cook-Swartz Doppler probe and flow couplers offer continuous monitoring. Wireless oximetry systems like ViOptix^®^ provide non-invasive, real-time perfusion data and support remote monitoring. **Conclusions**: Collectively, these innovations are transforming microsurgical breast reconstruction by increasing efficiency, consistency, and outcomes.

## Simple Summary

This review article explores how new technologies are improving microsurgical breast reconstruction, a procedure often used after mastectomy. The process involves four key steps: planning the surgical procedure, harvesting the tissue (termed free flap), connecting tiny blood vessels under the microscope, and monitoring blood supply to the free flap. Tools like CT scans and artificial intelligence improve surgical planning by identifying the best blood vessels to use. Robotic systems like the da Vinci and Symani^®^ help surgeons perform precise, less invasive operations, which protect muscles and enhance recovery. During surgery, imaging methods such as indocyanine green angiography help assess perfusion in real time. After surgery, advanced tools—including smartphone-based thermography, Doppler probes, and wearable sensors—allow for better and more convenient monitoring of blood supply to the reconstructed area. Overall, these advances make the procedure safer, faster, and more reliable, while also allowing for better outcomes and easier follow-up care, especially in outpatient or remote settings.

## 1. Introduction

More than 25 years after the first description of the deep inferior epigastric perforator (DIEP) flap, microsurgical breast reconstruction has become the gold standard for recreating the female breast after mastectomy [1]. Over time microsurgical breast reconstruction has been advancing rapidly with the integration of innovative technologies [2,3,4]. From preoperative perforator mapping enhanced by artificial intelligence to robotic platforms capable of submillimeter precision, the surgical landscape is evolving toward greater accuracy, efficiency, and patient safety [5,6,7].

Microsurgical breast reconstruction procedures can be divided into four main phases: preoperative flap planning, flap harvest, microsurgical anastomosis, and postoperative flap monitoring. Various imaging modalities have been described to enhance flap planning through preoperative perforator selection, thus increasing surgical efficiency [8,9]. Furthermore, minimally invasive, robotic-assisted or endoscopic flap harvest techniques have been described for selected patients with the advantage of limiting anterior rectus fascia incision, hence reducing donor site morbidity [10]. Moreover, microsurgical systems that reduce tremors, facilitate ergonomics and provide higher magnification have shown advantages in perforator vessel anastomoses or challenging locations [11]. Lastly, while historically flap monitoring relied on clinical parameters such as refill, color, and pencil Doppler, newer monitoring devices have allowed us to bury flaps completely in the context on nipple-sparing mastectomy, thus obviating the need for a skin paddle or are more sensitive in detecting minor changes in flap perfusion [12]. However, maintaining an overview of different technologies with their advantages and limitations is challenging.

This narrative review explores technological advances through the lens of the four critical phases of microsurgical breast reconstruction: flap planning, flap harvest, microvascular anastomosis, and postoperative flap monitoring. Drawing on both curated expert contributions from the «Aesthetic and Reconstructive Breast Surgery Network» (ARBS Network, https://www.arbsnetwork.com (access on 5 October 2025), Copyright 2025 Mark Allen Group, United Kingdom) website and a targeted literature search, the authors outline the most recent technological innovations shaping each step. By synthesizing developments in imaging modalities, artificial intelligence, robotic assistance, and real-time perfusion assessment, the authors aim to provide microsurgeons with a concise and focused overview of how technology is reshaping operative strategies and improving reconstructive outcomes. This narrative review furthermore highlights where the field stands today, identifies gaps in clinical evidence, and proposes future research directions.

## 2. Materials and Methods

To gather the most updated and relevant data, all content on the electronic database «Aesthetic and Reconstructive Breast Surgery Network» (ARBS Network, https://www.arbsnetwork.com, Copyright 2025 Mark Allen Group, UK) was screened regarding (micro-)surgical technology. The electronic database provides a curated collection of previous conference recordings of “London Breast Meeting” and “International Breast Symposium Düsseldorf”, among others, teaching webinars, as well as video content from various surgeons and industry.

The contributions were then grouped into one of four key phases where new technology has increased surgical precision, efficiency or safety: (1) flap planning, (2) flap harvest, (3) microvascular anastomosis, and (4) postoperative flap monitoring. For all contributions with a hyperlink provided, the video is available on demand for the readers after login. Literature by the presenting authors was added where available to consolidate the online content.

More references related to the content viewed were then searched on the bibliographic database MEDLINE (Bethesda, MD: U.S. National Library of Medicine) using database-specific subject headings and text words covering the technological concepts identified previously (last search 5 October 2025). No language or publication date restrictions were applied but conference abstracts were excluded from the search.

## 3. Results

**Figure 1:**
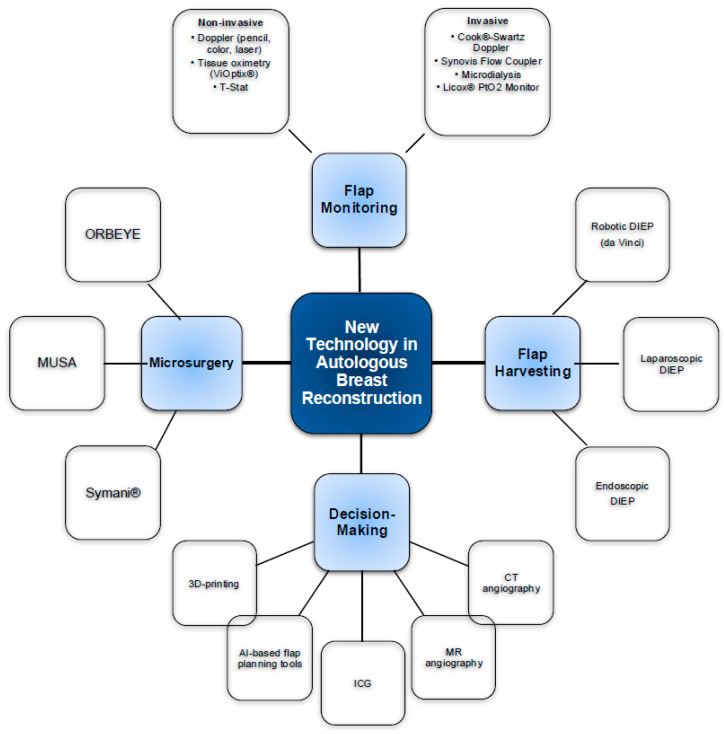
Overview of new technologies in microsurgical breast reconstruction.

24 contributions regarding new technology were identified on ARBS Network. Of these, 17 were relevant for this paper. An ordered list of all contributions is provided in Table 1. Figure 1 provides a concise overview of various technological innovations.

### 3.1. Flap Planning

Over time, various imaging modalities have been described to enhance flap planning through preoperative perforator selection, thus increasing surgical efficiency [8].

#### 3.1.1. CT Angiography (CTA)

Preoperative CTA has become a cornerstone in perforator flap breast reconstruction, enabling precise mapping of perforator location, caliber, and intramuscular trajectory to optimize flap design. A recent systematic review including 18 studies revealed that CTA reduces partial and total flap loss rates, while significantly shortening operative time in unilateral DIEP flaps compared to not using CTA [25]. A randomized, prospective trial further confirmed that CTA markedly decreases flap dissection and total operative time, particularly in bilateral reconstructions, without increasing complication rates [26]. The reduced operative time has substantial impacts on health-economics. Wade and colleagues estimated that shortening operative time by 21 min makes CTA “always cost-effective,” with potential UK savings of £0.5 million annually [25,27].

#### 3.1.2. MR Angiography (MRA)

MRA offers high-resolution, radiation-free visualization of perforator vessels, enabling accurate mapping of their location, size, and intramuscular trajectory for preoperative flap planning [28]. A retrospective study showed a mean reduction in operating time of 26 min for unilateral DIEP flap harvest and 40 min for bilateral harvest when preoperative, contrast-enhanced MRA was used, although these values were not statistically significant [29,30]. Compared with CTA, MRA avoids ionizing radiation and iodinated contrast, allowing safer repeat imaging and better depiction of intramuscular anatomy [31]. MRA furthermore provides superior soft-tissue contrast and multiplanar imaging, facilitating detailed visualization of perforator anatomy and supporting protocol optimization, reporting, and even early adoption of AI-assisted interpretation [28].

#### 3.1.3. Indocyanine Green (ICG) Angiography

Indocyanine green (ICG) angiography plays a critical role during flap planning and harvest in breast reconstruction by offering real-time, high-sensitivity visualization of tissue perfusion, thereby guiding intraoperative decision-making on perforator selection and flap design [32]. A systematic review found that ICG usage in free flap surgery is associated with a significant reduction in fat necrosis compared to clinical assessment alone, demonstrating its efficacy in predicting postoperative flap viability [32]. Intraoperative use of ICG during microvascular anastomosis additionally offers immediate visual confirmation of arterial and venous patency, with studies reporting reliable feedback and zero flap losses in the examined series [33]. Despite these benefits, variations in imaging protocol and lack of standardization—such as timing of perfusion assessment—underscore the need for optimized and reproducible methodologies to maximize clinical utility, as highlighted in a recent study [34].

#### 3.1.4. AI-Based Flap Planning Tools

Artificial intelligence (AI)–based tools are increasingly being integrated into preoperative flap planning for breast reconstruction, notably streamlining perforator identification by reducing image analysis time from several hours to under thirty minutes while maintaining comparable location accuracy in larger perforators [5]. These computer vision algorithms demonstrate high efficiency in processing CTA scans, particularly for perforators larger than 1.5 mm, though accuracy diminishes slightly for smaller vessels [5]. Beyond imaging, machine learning models are being applied prospectively to predict postoperative complications—such as flap failure—based on patient and procedural risk profiles, thus facilitating early identification of high-risk individuals [4].

#### 3.1.5. Three-Dimensional Printing

Patient-specific 3D-printed models have shown potential in improving visuo-spatial understanding of flap anatomy, thus enhancing clarity in dissection [35]. A study by DeFazio et al. compared 3D-printed models and interpretations of CTA imaging against operative findings [36]. 3D-printed models accurately matched intraoperative findings in perforator number, source vessel origin, and branching patterns, while CTA interpretations were less accurate. Another prospective study revealed a reduced intraoperative perforator identification time by over 7 min when using 3D-printed flap templates [37]. Lastly, according to a recent systematic review including eight studies, use of 3D-printed models reduced flap harvest time by up to 23 min per case [38]. However, model cost, production time as long as 18 h, and user adoption currently limit its implementation. A formal cost–benefit analysis in the context of microsurgical breast reconstruction is necessary for incorporated production cost, time savings and operating room (OR) costs.

### 3.2. Flap Harvest

Robotics were first introduced in breast reconstruction in 2006 by Boyd and colleagues for the harvest of intermammary recipient vessels in 20 patients [39]. A robotic approach to latissimus flap harvest was first described in 2011, followed by robotic DIEP flap harvest in 2018 [40]. Traditional DIEP flap harvest disrupts the abdominal wall to isolate perforators and obtain adequate pedicle length for microsurgical anastomosis. While lateral row perforator selection results in less intramuscular dissection, this approach puts motor nerves at higher risk of violation, leading to abdominal wall weakness, bulging, or hernia [41]. Robotic DIEP and endoscopic flap harvest minimize donor site morbidity by a much smaller anterior rectus fascial incision, a submuscular approach for pedicle dissection and by sparing motor nerves [40]. Table 2 provides an overview of benefits and limitations.

#### 3.2.1. Robotic-Assisted DIEP Flap Harvest Using Da Vinci Surgical System

Robotic-assisted DIEP flap harvest using the da Vinci surgical system (Intuitive Surgical, Inc., Sunnyvale, CA, USA) enables minimally invasive pedicle dissection with smaller anterior fascial incisions, thereby reducing donor-site morbidity such as hernia or abdominal bulge [7,21,42,45]. In a pioneering case report, the robot facilitated intra-abdominal dissection of the DIEP vessels through a 1.5 cm fascial incision, yielding no postoperative complications at nine-month follow-up [45]. A retrospective series of four patients undergoing robotic-assisted DIEP harvest demonstrated successful bilateral flap reconstructions with no flap failures or abdominal wall morbidity [42]. Cadaveric feasibility studies comparing transabdominal preperitoneal (TAPP) and totally extraperitoneal (TEP) approaches reported mean harvest times of 56 min for TAPP and 65 min for TEP, with TEP preserving the posterior rectus sheath and reducing intra-abdominal manipulation [46]. A recent literature review of 56 robotic-assisted DIEP procedures confirmed consistent safety and favorable aesthetic outcomes, though noted increased operative time and cost as limitations [47]. Most recently, a cohort of 23 patients (46 flaps) undergoing bilateral robotic DIEP harvest using the da Vinci Xi, combined with ICG fluorescence guidance, achieved mean fascial incisions of 4.1 cm and no intra-abdominal injuries, with an average hospital stay under 4 days [48]. In 2021, Choi et al. described the first extraperitoneal DIEP harvest using the da Vinci SP system by placing the single robot port through the neo-umbilicus. This method conserves the posterior rectus sheath and does not enter the peritoneal cavity, thereby further reducing the risks associated with abdominal surgery [49].

#### 3.2.2. Endoscopic DIEP (Ediep) and Total Extraperitoneal Laparoscopic (TEP-Lap) Flap Harvest

A cadaveric study conducted by Stroumza et al. emphasized that eDIEP could offer improved precision and reduced postoperative complications [18]. Another retrospective study that compared endoscopic and TEP-lap incisions found TEP-lap lengths of fascial incisions to be shorter than endoscopic incisions (2.0 ± 0.6 cm vs. 4.5 ± 0.5 cm, (*p* < 0.0001) [50]). No subjects required conversion to an open harvest. There were no bleeding complications, intra-abdominal injuries, flap losses, or abdominal bulges/hernias. A recent case series involving nine patients with endoscopic DIEP harvest reported an uneventful postoperative recovery in all cases [51]. A potential advantage of endoscopic flap harvest over robotic-assisted harvest might be the overall reduced cost [50].

### 3.3. Microsurgical Anastomosis

Over the past two decades, the use of robotic systems in surgery has expanded to include complex microsurgical tasks. However, the adoption of this technology has also raised critical questions about efficiency, learning curve, and outcomes when compared with traditional manual anastomotic techniques. A recent systematic review provides an overview of the current landscape of robotic-assisted microsurgery [52]. A direct comparison of robotic microsurgery platforms is provided in Table 3.

#### 3.3.1. Symani^®^ Surgical System

The Symani^®^ robotic platform (Medical Microinstruments, Inc., Wilmington, Delaware, United States) enhances surgical precision by offering tremor filtration, motion scaling, and 3D visualization. In a preclinical study, patency rates were 100% during the day of surgery for all anastomoses and stayed at 100% 28 days after surgery for the robotic subgroups [53]. Clinical studies have demonstrated that the Symani^®^ system facilitates rapid and precise in-flap anastomoses, with arterial and venous anastomoses completed in 23 and 28 min, respectively, using 9/0 nylon sutures, without intraoperative complications [54]. The system’s ergonomic design also reduces surgeon fatigue during lengthy procedures. A retrospective analysis by Wessel et al. encompassed 28 patients who underwent unilateral robotic-assisted autologous breast reconstruction [55]. The procedures utilized a combined approach involving the Symani^®^ Surgical System and the RoboticScope, focusing on precision in microvascular anastomoses. The study reported an average arterial anastomosis duration of 29 min and a flap survival rate of 100%, indicating the system’s efficacy in enhancing surgical outcomes. In a case series exploring the miraDIEP concept, the Symani^®^ platform furthermore allowed minimally invasive DIEP harvest with a short pedicle through an anterior fascial incision as short as 2.5 cm with prepectoral anastomosis to an internal mammary artery perforator (average IMA perforator diameter 1.14 mm) [56].

#### 3.3.2. MUSA Platform

While there is no published study specifically using the MUSA platform (Microsure B.V., Eindhoven, The Netherlands) for breast reconstruction, the existing MUSA research focuses on lymphaticovenous anastomosis (LVA) in the context of breast cancer-related lymphedema. MUSA is a small, table-mounted microsurgical robot designed to filter surgeon tremor and enable motion scaling. A first-in-human randomized pilot trial demonstrated that robot-assisted LVA using MUSA is feasible and safe, with comparable one-year outcomes—including quality of life improvements and anastomosis patency—when compared to manual surgery [57]. The system integrates with conventional microscopes and standard instruments without disrupting surgical workflow, though initial anastomosis times were longer but showed a steep learning curve [58].

#### 3.3.3. Venous Coupler

Venous couplers have become integral to microvascular breast reconstruction, streamlining venous anastomosis and enhancing surgical efficiency. A study by O’Connor and colleagues demonstrated that using a venous coupler significantly reduced operating times and the need for return-to-theatre procedures, with a coupler failure rate of 1.4% compared to 3.57% for hand-sewn venous anastomoses (*p* = 0.001). This reduction in complications translates to cost savings and improved patient outcomes [59]. The size of the coupler plays a critical role in the success of venous anastomoses. Broer et al. found that using couplers smaller than 2.5 mm was associated with increased risks of venous insufficiency, fat necrosis, and the need for fat grafting in autologous breast reconstruction. Therefore, selecting an appropriately sized coupler is essential for minimizing complications and optimizing flap survival [60].

#### 3.3.4. ORBEYE

The ORBEYE 3D digital microscope enhances microsurgical breast reconstruction by providing high-definition, stereoscopic imaging that improves visualization during microvascular anastomoses. Chang et al. highlighted its ergonomic design, which reduces surgeon fatigue and potentially increases surgical efficiency [24]. The system’s ability to project the surgical field on large monitors facilitates better team communication and coordination.

### 3.4. Flap Monitoring

Postoperative flap monitoring allows early detection of clinical complications, and timely re-operation can prevent the need for extensive correction procedures, thus reducing healthcare costs, hospitalization time, and most importantly, psychological burden for patients. Flap monitoring can increase flap survival rate to 95%. A recent comprehensive review provides a more detailed overview of different flap monitoring technologies [61]. True positive rates of new technologies to detect flap compromise are listed in Table 4 [12].

#### 3.4.1. Non-Invasive Methods

- Doppler systems (pencil, color, laser)

Breast flap monitoring using Doppler systems—such as pencil probes, color Doppler, and laser Doppler flowmetry—plays a crucial role in detecting early signs of vascular compromise, thereby enhancing surgical outcomes. Studies have demonstrated that laser Doppler flowmetry offers real-time, non-invasive assessment of tissue perfusion, allowing for timely interventions in cases of compromised flaps. A study by Hölzle et al. (2006) highlighted the effectiveness of simultaneous non-invasive laser Doppler flowmetry and tissue spectrophotometry in monitoring free flaps, noting its utility in detecting early signs of flap failure [62]. Additionally, pencil Doppler probes have been widely utilized to assess perforator vessel flow during flap planning, aiding in the selection of optimal perforators for flap harvest.

- ViOptix^®^

The ViOptix^®^ Tissue Oximeter is a noninvasive monitoring device that utilizes near-infrared spectroscopy to assess tissue oxygen saturation (StO_2_) in free flap breast reconstruction. It penetrates tissue up to 4–8 mm. A study by Keller demonstrated that ViOptix^®^ effectively detected ischemic events during flap transfer and identified venous thrombosis before clinical signs were apparent, allowing for timely interventions and flap salvage [63]. In a larger cohort, Keller introduced a diagnostic algorithm using StO_2_ measurements to predict vascular compromise, achieving high diagnostic accuracy within one hour of occlusive events [64]. Additionally, Schoenbrunner et al. conducted a cost-effectiveness analysis comparing ViOptix^®^ with clinical examination alone, finding that while ViOptix^®^ slightly improved effectiveness, it was not cost-effective under typical pricing models [65]. These findings suggest that ViOptix^®^ enhances flap monitoring by providing early detection of vascular issues, potentially improving surgical outcomes. However, considerations regarding cost-effectiveness are important when integrating this technology into clinical practice.

- T-Stat

T-Stat uses reflectance spectroscopy to measure tissue oxygen saturation (StO_2_) at a superficial depth (1–2 mm), primarily reflecting capillary and superficial dermal perfusion. In a prospective clinical trial, T-Stat was found to be more sensitive than Doppler or clinical examination for early detection of flap compromise [66]. The study suggests T-Stat has higher sensitivity and reasonable specificity compared with intermittent methods.

#### 3.4.2. Invasive Methods

- Cook-Swartz Doppler probe

The Cook-Swartz Doppler probe is a widely utilized implantable device for postoperative monitoring of free flap breast reconstructions. A study by Smit et al. involving 145 probes in 121 microvascular breast reconstructions reported a low complication rate, with only 2 complete flap losses and 15 instances of audible signal issues, all of which were promptly addressed [67]. This highlights the device’s reliability and the surgical team’s responsiveness in managing potential complications. In a comparative analysis, Um and colleagues found no statistically significant differences in outcomes between the Cook-Swartz Doppler and the Synovis GEM™ Flow Coupler in free flap breast reconstruction [68]. Both devices demonstrated comparable false-positive and false-negative rates, as well as similar flap survival rates, suggesting that the choice between these devices may be based on surgeon preference and institutional protocols. Rozen et al. conducted a study comparing clinical monitoring with the Cook-Swartz Doppler probe in 547 consecutive free flaps. The results indicated that the Doppler probe improved flap salvage rates without increasing false-positive takeback rates, underscoring its effectiveness as an adjunct to traditional monitoring methods [69]. In a further prospective comparative study, each of the Cook-Swartz Doppler probe, microdialysis and clinical assessment was found suitable for monitoring in free tissue transfer [70]. In a systematic review, Agha et al. assessed the efficacy of the Cook-Swartz Doppler in detecting free-flap compromise. The review found that the Doppler probe had high sensitivity and specificity, making it a valuable tool for early detection of vascular issues in free flaps [71].

- Synovis GEM™ flow coupler

In addition to improving surgical efficiency, venous couplers facilitate postoperative monitoring. Chadwick et al. reported that the Synovis GEM™ flow coupler effectively monitored buried free flaps in breast reconstruction, eliminating the need for additional procedures to remove skin paddles [72]. This capability enhances patient comfort and reduces the risk of unnoticed flap compromise, underscoring the multifaceted benefits of venous couplers in reconstructive surgery.

- Microdialysis

Microdialysis offers real-time biochemical analysis of tissue metabolism. A study by Udesen et al. demonstrated that microdialysis could detect ischemia in free TRAM flaps at an early stage, allowing for prompt surgical intervention [73]. Similarly, Whitaker and colleagues conducted a multicenter comparison of 398 free flaps and found that microdialysis provided objective data on tissue perfusion, aiding in the early identification of compromised flaps [74].

- LICOX^®^

The LICOX^®^ system is an advanced microdialysis tool that continuously measures tissue oxygen tension (ptiO_2_) and lactate levels, providing real-time metabolic data to assess flap viability in breast reconstruction. A study by Hirigoyen et al. demonstrated that continuous ptiO_2_ monitoring with the LICOX^®^ catheter effectively detected early signs of ischemia in free flaps, enabling timely surgical intervention and improving flap survival rates [75]. However, a prospective observational study that compared LICOX^®^ with near-infrared spectroscopy or bedside monitoring in buried flaps did not find LICOX^®^ to be cost-efficient [76].

## 4. Discussion

This narrative review synthesizes the most recent technological advancements across four critical phases—flap planning, flap harvest, microvascular anastomosis, and postoperative flap monitoring—demonstrating how technology is reshaping clinical practice and addressing challenges in autologous breast reconstruction. This narrative review has some strengths. By sourcing data from ARBS Network, the authors could identify hitherto unpublished results. Also, the available videos might provide valuable information for the interested reader. However, while many new technologies could be identified by screening the online database, this review is not complete. Identifying all possible state-of-the-art technologies as part of a systematic review was beyond the scope of this article but could be part of a future research project. Furthermore, we did not aim at providing exhaustive descriptions of the different technologies but rather a succinct overview of relevant data. More information can be found in the referenced literature or web links provided.

Various flap preoperative planning tools have helped visualize perforator anatomy, thus shortening operative time [8]. However, high-quality, multicenter RCTs are needed to compare CTA directly with alternative modalities such as MRA or combined ICG fluorescence. Integration of AI for automated perforator identification and development of consensus imaging protocols will standardize practice and reduce variability [77]. Economic evaluations over a 5–10-year horizon, including cost per quality-adjusted life year, are vital for policy decisions [25].

Robotic and endoscopic flap harvest techniques offer the advantage of reduced anterior rectus sheath incision, improved perforator visualization and improved ergonomics [40]. Compared with traditional harvest techniques, these techniques have shown to be safe with comparable flap loss rates. However, higher cost and surgeon as well as OR staff learning curve currently limit their implementation.

New microsurgical platforms such as MUSA and Symani^®^ allow supermicrosurgical anastomosis with tremor filtration, motion scaling and improved ergonomics, thereby opening microsurgery to a wider surgical community. Anastomotic patency and flap loss rates have shown to be similar compared with traditional techniques [52]. Their application is limited by acquisition and maintenance costs.

Adjunctive flap monitoring technologies have been associated with reduced flap loss rates compared with clinical monitoring alone, and invasive methods offer to monitor buried free flaps. However, increased cost and staff learning curves may limit their application [12].

Surgeon education in the setting of new technologies remains a challenge. As such, there are no standardized training programs for robotic plastic surgery, and robotic training has not yet been integrated into most plastic surgery residency curricula. Robotic flap harvest is not widely practiced, contributing to its slow adoption and the limited availability of large-scale outcome data. Moreover, learning curves and capital costs associated with robotic and endoscopic systems may limit accessibility, particularly in resource-constrained settings [78]. By providing recorded video content, online platforms such as ARBS Network can serve as an educational adjunct for technological innovations. However, hands-on training and simulator modules are essential and currently scarce.

Looking ahead, artificial intelligence is poised to play an increasingly integral role throughout the reconstructive pathway. Beyond image analysis, AI-driven predictive analytics could enable personalized risk stratification, guiding perioperative management to reduce complications. The fusion of AI with robotic platforms may facilitate augmented intraoperative decision-making through real-time feedback and adaptive control, enhancing surgical precision. Additionally, machine learning algorithms could optimize postoperative monitoring by integrating multimodal sensor data to improve early detection of flap compromise.

## 5. Conclusions and Future Directions

In conclusion, the rapid integration of imaging advancements, robotic and endoscopic techniques, precision microsurgical tools, and intelligent monitoring systems is transforming microsurgical breast reconstruction. These technologies collectively improve surgical accuracy, reduce morbidity, and enhance flap survival, contributing to superior patient outcomes. Continued multidisciplinary collaboration, rigorous clinical research, and integrating these technologies into plastic surgery teaching programs will be essential to fully realize the potential of these innovations and to democratize access to cutting-edge reconstructive care in the near future.

## Figures and Tables

**Table 1:** Overview of all content identified on ARBS Network related to new technology in autologous breast reconstruction. Where available, bibliographic references are provided.

Area	Author/Speaker	Title	Innovation	Hyperlink	Reference
Flap planning	J. Masia	Decision making with ICG technology for autologous breast reconstruction	ICG technology	https://www.arbsnetwork.com/content/conferences/decision-making-with-icg-technology-for-autologous-breast-reconstruction (accessed on 5 October 2025)	
Flap planning	N. Karunanithy	Video Workshop Session: Imaging for Microsurgeons	CTA	https://www.arbsnetwork.com/content/conferences/imaging-and-the-choice-of-the-one-perforator-in-autologous-breast-reconstruction (accessed on 5 October 2025)	
Flap planning	P. Blondeel	Imaging and the choice of the ONE perforator in autologous breast reconstruction	ICG technology Thermographic camera	https://www.arbsnetwork.com/content/conferences/imaging-and-the-choice-of-the-one-perforator-in-autologous-breast-reconstruction (accessed on 5 October 2025)	[13]
Flap planning	R. Allen	Imaging and the choice of the ONE perforator in autologous breast reconstruction	CTA MRA	https://www.arbsnetwork.com/content/conferences/imaging-and-the-choice-of-the-one-perforator-in-autologous-breast-reconstruction (accessed on 5 October 2025)	[14]
Flap planning	S. Allen	Do plastic surgeons need to be certified in preoperative imaging?	CTA	https://www.arbsnetwork.com/content/conferences/do-plastic-surgeons-need-to-be-certified-in-preoperative-imaging (accessed on 5 October 2025)	
Flap planning	N. Karunanithy	Adjuncts to autologous reconstruction	CTA	https://www.arbsnetwork.com/content/conferences/adjuncts-to-autologous-reconstruction (accessed on 5 October 2025)	
Flap planning	G. Pons	Refinement of autologous breast reconstruction	CTA MRA ICG technology	https://www.arbsnetwork.com/content/conferences/refinement-of-autologous-breast-reconstruction (accessed on 5 October 2025)	[15,16]
Flap harvest	M. Nahabedian, H. Sbitani	Webinar: New technologies for improving breast surgery	Robotic-assisted DIEP harvest	https://www.arbsnetwork.com/content/webinars/webinar-new-technologies-for-improving-breast-surgery (accessed on 5 October 2025)	[17]
Flap harvest	M. Atlan	Innovation in autologous flaps: Endoscopic DIEP flap	Endoscopic DIEP harvest	https://www.arbsnetwork.com/content/conferences/innovation-in-autologous-flaps-endoscopic-diep-flap (accessed on 5 October 2025)	[18]
Flap harvest	J. J. Huang	Artificial intelligence and robotics	Robotic-assisted DIEP harvest	https://www.arbsnetwork.com/content/conferences/artificial-intelligence-and-robotics (accessed on 5 October 2025)	[19]
Flap harvest	J. Selber	Robotic surgery in breast and microsurgery	Robotic-assisted DIEP harvest	https://www.arbsnetwork.com/content/conferences/robotic-surgery-in-breast-and-microsurgery (accessed on 5 October 2025)	[20,21]
Microsurgery	M. Innocenti	Artificial intelligence and robotics	Symani^®^	https://www.arbsnetwork.com/content/conferences/artificial-intelligence-and-robotics (accessed on 5 October 2025)	[22,23]
Microsurgery	M. Innocenti	Robotic surgery in breast and microsurgery	Symani^®^	https://www.arbsnetwork.com/content/conferences/robotic-surgery-in-breast-and-microsurgery (accessed on 5 October 2025)	[22,23]
Microsurgery	E. I. Chang	Innovations in autologous reconstruction	ORBEYE	https://www.arbsnetwork.com/content/conferences/innovations-in-autologous-reconstruction (accessed on 5 October 2025)	[24]
Monitoring	S. Suominen	Flap monitoring in autologous reconstruction: short and sweet	Laser Doppler Color Doppler pO2 (ViOptix^®^) Thermocamera ICG pO2 (LICOX^®^) Microdialysis Implantable laser Doppler Flow coupler	https://www.arbsnetwork.com/content/conferences/flap-monitoring-in-autologous-reconstruction-short-and-sweet (accessed on 5 October 2025)	
Monitoring	M. Chrysopoulo	T-Stat symposium: Flap salvage-the earliest possible detection	T-Stat Tissue Oximeter	https://www.arbsnetwork.com/content/conferences/t-stat-symposium-flap-salvage-the-earliest-possible-detection (accessed on 5 October 2025)	
Monitoring	C. Andree	Innovations in autologous reconstruction	Flow coupler Cook-Swartz Doppler Microdialyisis	https://www.arbsnetwork.com/content/conferences/innovations-in-autologous-reconstruction (accessed on 5 October 2025)	

Abbreviations: CTA, CT-angiography; DIEP, deep inferior epigastric perforator; ICG, infracyanine green; MRA, MR-angiography.

**Table 2:** Comparison of different flap harvesting technologies [40].

Technology	Advantages	Disadvantages
Robotic DIEP (da Vinci)	Perforator dissection with high-resolution optics [20] Superior ergonomics Shorter hospital LOS Decreased opioid requirements [42] Increased patient satisfaction [43] Decreased donor site morbidity by limiting anterior rectus sheath incision	Currently Off-Label by the U.S. Food and Drug Administration Cost for acquisition and maintenance [44] Learning curve for the surgeon and OR staff
Endoscopic DIEP	Lower cost than robotic harvest Decreased donor site morbidity by limiting anterior rectus sheath incision	Learning curve for the surgeon and OR staff Cost for acquisition and maintenance

**Table 3:** Comparison of different microsurgery platforms.

Technology	Manufacturer (Country)	Advantage	Disadvantage
Symani^®^	MMI (Italy)	Tremor filtration Motion scaling Enabling supermicrosurgery	Cost for acquisition and maintenance Separate instruments necessary Large size Use for anastomosis only; no possibility for dissection
MUSA	Microsure (The Netherlands)	Tremor filtration Motion scaling Standard microsurgical instruments can be used Small size	Cost for acquisition and maintenance

**Table 4:** Comparison of different flap monitoring technologies (based on [12]).

Monitoring Technology	Invasiveness	True-Positive Rate Among Flap Take Backs	Salvage Rate	Flap Failure Rate	Disadvantage
Clinical exam	Non-invasive	61.9%	77.4%	5.1%	Requiring external skin paddle
ViOptix^®^	Non-invasive	74.76%	88.62%	2.5%	Requiring external skin paddle, higher cost, learning curve for staff
T-Stat	Non-invasive	100%	100%	0%	Requiring external skin paddle, higher cost, learning curve for staff
Cook-Swartz Doppler	Invasive	80.17%	83.63%	2.6%	Higher cost
Synovis GEM™ flow coupler	Invasive	90%	82%	2.8%	Higher cost

## Data Availability

No new data were created or analyzed in this study. Data sharing is not applicable to this article.

## Abbreviations

The following abbreviations are used in this manuscript:

ARBS Network	Aesthetic and Reconstructive Breast Surgery Network
AI	Artificial Intelligence
CTA	CT angiography
DIEP	Deep inferior epigastric perforator
ICG	Indocyanine green
LVA	Lymphaticovenous anastomosis
LOS	Length of stay
MRA	MR-angiography
OR	Operating room
ptiO_2_	Tissue oxygen tension
StO_2_	Tissue oxygen saturation
TAPP	Transabdominal preperitoneal
TEP-lap	Total extraperitoneal laparoscopic
TRAM	Transverse rectus abdominis myocutaneous

## References

[1] Allen R.J., Treece P. (1994). Deep Inferior Epigastric Perforator Flap for Breast Reconstruction. Ann. Plast. Surg..

[2] Cho M.-J., Schroeder M., Garcia J.F., Royfman A., Moreira A. (2025). The Current State of the Art in Autologous Breast Reconstruction: A Review and Modern/Future Approaches. J. Clin. Med..

[3] Speck N.E., Grufman V., Farhadi J. (2022). Trends and Innovations in Autologous Breast Reconstruction. Arch. Plast. Surg..

[4] Allam O., Foster C., Knoedler L., Knoedler S., Oh S.J., Pomahac B., Ayyala H.S. (2024). Future of autologous breast reconstruction: A review of novel technological innovations. Plast. Aesthetic Res..

[5] Cevik J., Seth I., Rozen W.M. (2023). Transforming breast reconstruction: The pioneering role of artificial intelligence in preoperative planning. Gland. Surg..

[6] Burns H.R., McLennan A., Xue E.Y., Yu J.Z., Selber J.C. (2024). Robotics in Microsurgery and Supermicrosurgery. Semin. Plast. Surg..

[7] Tanna N.M., Sugiyama G., Smith M.L., Sanchez S.B., Minasian R.A., Robinson E.B., Silverman J.B., Shuck J.W., Selber J.M. (2023). The Full Continuum of Robotic Breast Surgery: Robotic-assisted Mastectomy, Robotic DIEP Flap, and Robotic Supermicrosurgery. Plast. Reconstr. Surg.–Glob. Open.

[8] Kiely J., Kumar M., Wade R.G. (2021). The accuracy of different modalities of perforator mapping for unilateral DIEP flap breast reconstruction: A systematic review and meta-analysis. J. Plast. Reconstr. Aesthetic Surg..

[9] Rodkin B., Hunter-Smith D.J., Rozen W.M. (2019). A review of visualized preoperative imaging with a focus on surgical procedures of the breast. Gland. Surg..

[10] Yusufov S., Startseva O., Khalfaoui S., Zhigailova E., Gabriyanchik M., Manasherova D., Meskhi K., Reshetov I. (2025). Robot-Assisted Versus Conventional Harvesting of DIEP and Latissimus Dorsi Flaps for Breast Reconstruction in Post-Mastectomy Women: A Systematic Review and Meta-Analysis. J. Clin. Med..

[11] Brown H., Brown R.A., Lenkiu L., SamSam A., Lopez J., Sawh-Martinez R. (2025). Robotic-assisted Supermicrosurgery in Plastic Surgery: A Systematic Literature Review. Plast. Reconstr. Surg.–Glob. Open.

[12] Lacey H., Kanakopoulos D., Hussein S., Moyasser O., Ward J., King I. (2023). Adjunctive technologies in postoperative free-flap monitoring: A systematic review. J. Plast. Reconstr. Aesthetic Surg..

[13] Zötterman J., Opsomer D., Farnebo S., Blondeel P., Monstrey S., Tesselaar E. (2020). Intraoperative Laser Speckle Contrast Imaging in DIEP Breast Reconstruction: A Prospective Case Series Study. Plast. Reconstr. Surg.–Glob. Open.

[14] Canizares O., Mayo J., Soto E., Allen R.J., Sadeghi A. (2015). Optimizing Efficiency in Deep Inferior Epigastric Perforator Flap Breast Reconstruction. Ann. Plast. Surg..

[15] Masia J., Clavero J., Larrañaga J., Alomar X., Pons G., Serret P. (2006). Multidetector-row computed tomography in the planning of abdominal perforator flaps. J. Plast. Reconstr. Aesthetic Surg..

[16] Masia J., Navarro C., Clavero J.A., Alomar X. (2011). Noncontrast Magnetic Resonance Imaging for Preoperative Perforator Mapping. Clin. Plast. Surg..

[17] Nahabedian M.Y. (2023). The deep inferior epigastric perforator flap: Where we started and where we are now. Gland. Surg..

[18] Stroumza N., Barthelemy R.N., Majoulet L., Delchet O., Qassemyar Q., Atlan M. (2017). Endoscopic DIEP flap dissection (eDIEP): An experimental cadaveric study. J. Plast. Reconstr. Aesthetic Surg..

[19] Kuo W.-L., Wong A.W.-J., Tsai C.-Y., Chen Y.-F., Chang T.N.-J., Cheong D.C.-F., Huang J.-J.M. (2025). Oncoplastic Entirely Robot-Assisted Approach: Incorporating Robotic Surgery in Both Mastectomy and DIEP Flap Reconstruction. Plast. Reconstr. Surg..

[20] Selber J.C. (2020). The Robotic DIEP Flap. Plast. Reconstr. Surg..

[21] Bishop S.N., Selber J.C. (2021). The RoboDIEP: Robotic-Assisted Deep Inferior Epigastric Perforator Flaps for Breast Reconstruction. Robotics in Plastic and Reconstructive Surgery.

[22] Innocenti M. (2022). Back to the Future: Robotic Microsurgery. Arch. Plast. Surg..

[23] Teichmann H., Innocenti M. (2021). Development of a New Robotic Platform for Microsurgery. Robotics in Plastic and Reconstructive Surgery.

[24] Ahmad F.I., Mericli A.F., DeFazio M.V., Chang E.I., Hanasono M.M., Pederson W.C., Kaufman M., Selber J.C. (2020). Application of the ORBEYE three-dimensional exoscope for microsurgical procedures. Microsurgery.

[25] Rupra R.S., Ruccia F., Daneshi K., Aftab F., Yousif Y.F., Khan G.R., Dehnadi S., AlSaidi Y.H., Maggialetti N., Lorusso G. (2025). A systematic review and meta-analysis on computed tomography angiography mapping for deep inferior epigastric perforator flap breast reconstruction. Front. Oncol..

[26] Colakoglu S., Tebockhorst S., Freedman J., Douglass S., Siddikoglu D., Chong T.W., Mathes D.W. (2022). CT angiography prior to DIEP flap breast reconstruction: A randomized controlled trial. J. Plast. Reconstr. Aesthetic Surg..

[27] Wade R.G., Watford J., Wormald J.C., Bramhall R.J., Figus A. (2018). Perforator mapping reduces the operative time of DIEP flap breast reconstruction: A systematic review and meta-analysis of preoperative ultrasound, computed tomography and magnetic resonance angiography. J. Plast. Reconstr. Aesthetic Surg..

[28] Thimmappa N.D. (2024). MRA for Preoperative Planning and Postoperative Management of Perforator Flap Surgeries: A Review. J. Magn. Reson. Imaging.

[29] Schaverien M.V., Ludman C.N., Neil-Dwyer J., Perks G.B., Akhtar N., Rodrigues J.N., Benetatos K., Raurell A., Rasheed T., McCulley S.J. (2011). Contrast-Enhanced Magnetic Resonance Angiography for Preoperative Imaging in DIEP Flap Breast Reconstruction. Plast. Reconstr. Surg..

[30] Schaverien M.V., McCulley S.J. (2016). Contrast-Enhanced Magnetic Resonance Angiography for Preoperative Imaging in DIEP Flap Breast Reconstruction. Breast Reconstruction.

[31] Thimmappa N., Bhat A.P., Bishop K., Nagpal P., Prince M.R., Saboo S.S. (2019). Preoperative cross-sectional mapping for deep inferior epigastric and profunda artery perforator flaps. Cardiovasc. Diagn. Ther..

[32] Parmeshwar N., Sultan S.M., Kim E.A., Piper M.L. (2021). A Systematic Review of the Utility of Indocyanine Angiography in Autologous Breast Reconstruction. Ann. Plast. Surg..

[33] Bombardelli J., Farhat S., Hagopian A.D.L.F., Hua J., Schusterman M.A.I., Echo A. (2023). Evaluation of Intraoperative Anastomotic Patency with Angiography in Microsurgical Breast Reconstruction. Plast. Reconstr. Surg.–Glob. Open.

[34] Nguyen C.L., Dayaratna N., Easwaralingam N., Seah J.L., Azimi F., Mak C., Pulitano C., Warrier S.K. (2025). Developing an Indocyanine Green Angiography Protocol for Predicting Flap Necrosis During Breast Reconstruction. Surg. Innov..

[35] Mehta S., Byrne N., Karunanithy N., Farhadi J. (2016). 3D printing provides unrivalled bespoke teaching tools for autologous free flap breast reconstruction. J. Plast. Reconstr. Aesthetic Surg..

[36] DeFazio M.V., Arribas E.M., Ahmad F.I., Le-Petross H.T., Liu J., Chu C.K., Santiago L., Clemens M.W. (2020). Application of Three-Dimensional Printed Vascular Modeling as a Perioperative Guide to Perforator Mapping and Pedicle Dissection during Abdominal Flap Harvest for Breast Reconstruction. J. Reconstr. Microsurg..

[37] Chae M.P., Hunter-Smith D.J., Chung R.D., Smith J.A., Rozen W.M. (2021). 3D-printed, patient-specific DIEP flap templates for preoperative planning in breast reconstruction: A prospective case series. Gland. Surg..

[38] Ghasroddashti A., Guyn C., Martou G., Edmunds R.W. (2024). Utility of 3D-printed vascular modeling in microsurgical breast reconstruction: A systematic review. J. Plast. Reconstr. Aesthetic Surg..

[39] Boyd B., Umansky J., Samson M., Boyd D., Stahl K. (2006). Robotic Harvest of Internal Mammary Vessels in Breast Reconstruction. J. Reconstr. Microsurg..

[40] Hammond J.B., Egan K.G., Selber J.C. (2024). Robotic approaches to breast reconstruction. Plast. Aesthetic Res..

[41] Elver A.A., Matthews S.A., Egan K.G., Bowles E.L., Nazir N., Flurry M., Holding J., Lai E.C., Butterworth J.A. (2023). Characterizing Outcomes of Medial and Lateral Perforators in Deep Inferior Epigastric Perforator Flaps. J. Reconstr. Microsurg..

[42] Daar D.A., Anzai L.M., Vranis N.M., Schulster M.L., Frey J.D., Jun M., Zhao L.C., Levine J.P. (2022). Robotic deep inferior epigastric perforator flap harvest in breast reconstruction. Microsurgery.

[43] Lee M.J., Won J., Song S.Y., Park H.S., Kim J.Y., Shin H.J., Kwon Y.I., Lee D.W., Kim N.Y. (2022). Clinical outcomes following robotic versus conventional DIEP flap in breast reconstruction: A retrospective matched study. Front. Oncol..

[44] Williams S.B., Prado K., Hu J.C. (2014). Economics of Robotic Surgery. Urol. Clin. N. Am..

[45] Gundlapalli V.S., Ogunleye A.A., Scott K., Wenzinger E., Ulm J.P., Tavana L., Pullatt R.C., Delaney K.O. (2018). Robotic-assisted deep inferior epigastric artery perforator flap abdominal harvest for breast reconstruction: A case report. Microsurgery.

[46] Bustos S.S., Manrique O.J., Mohan A.T., Nguyen M.-D., Martinez-Jorge J., Forte A.J., Terzic A. (2020). Robotic-Assisted DIEP Flap Harvest for Autologous Breast Reconstruction: A Comparative Feasibility Study on a Cadaveric Model. J. Reconstr. Microsurg..

[47] Khan M.T.D., Won B.W., Baumgardner K.B., Lue M.B., Montorfano L., Hosein R.C., Wang H.T., Martinez R.A. (2022). Literature Review. Ann. Plast. Surg..

[48] Murariu D., Chen B., Bailey E., Nelson W., Fortunato R., Nosik S., Moreira A. (2025). Transabdominal Robotic Harvest of Bilateral DIEP Pedicles in Breast Reconstruction: Technique and Interdisciplinary Approach. J. Reconstr. Microsurg..

[49] Choi J.H., Song S.Y.M., Park H.S.M., Kim C.H., Kim J.Y., Lew D.H.M., Roh T.S.M., Lee D.W.M. (2021). Robotic DIEP Flap Harvest through a Totally Extraperitoneal Approach Using a Single-Port Surgical Robotic System. Plast. Reconstr. Surg..

[50] Shakir S., Spencer A.B., Piper M., Kozak G.M., Soriano I.S., Kanchwala S.K. (2021). Laparoscopy allows the harvest of the DIEP flap with shorter fascial incisions as compared to endoscopic harvest: A single surgeon retrospective cohort study. J. Plast. Reconstr. Aesthetic Surg..

[51] Katsuragi R. (2025). Endoscope-Assisted DIEP Flap for Breast Reconstruction: A Consecutive Series of Nine Cases. Indian J. Plast. Surg..

[52] Cannizzaro D., Scalise M., Zancanella C., Paulli S., Peron S., Stefini R. (2024). Comparative Evaluation of Major Robotic Systems in Microanastomosis Procedures: A Systematic Review of Current Capabilities and Future Potential. Brain Sci..

[53] Menichini G., Malzone G., Tamburello S., Andreoli A.L., Mori F., Ballestín A., Shiraki T. (2024). Safety and efficacy of Symani robotic-assisted microsurgery: Assessment of vascular anastomosis patency, thrombus, and stenosis in a randomized preclinical study. J. Plast. Reconstr. Aesthetic Surg..

[54] Vollbach F.H., Bigdeli A.K., Struebing F., Weigel J.L., Gazyakan E., Kneser U. (2024). Using a Microsurgical Robotic Platform for In-flap Anastomosis in Autologous Bipedicular Breast Reconstruction. Plast. Reconstr. Surg.–Glob. Open.

[55] Wessel K.J., Varnava C., Wiebringhaus P., Hiort M., Hirsch T., Kückelhaus M. (2024). Roboter-assistierte Mikrochirurgie zur autologen Brustrekonstruktion. Handchir. · Mikrochir. · Plast. Chir..

[56] Kueckelhaus M.M. (2024). Minimally Invasive Robotic-assisted Perforator-to-Perforator DIEP Flap Breast Reconstruction. Plast. Reconstr. Surg.–Glob. Open.

[57] van Mulken T.J.M., Schols R.M., Scharmga A.M.J., Winkens B., Cau R., Schoenmakers F.B.F., Qiu S.S., van der Hulst R.R.W.J., Keuter X.H.A., MicroSurgical Robot Research Group (2020). First-in-human robotic supermicrosurgery using a dedicated microsurgical robot for treating breast cancer-related lymphedema: A randomized pilot trial. Nat. Commun..

[58] van Mulken T.J.M., Wolfs J.A.G.N., Qiu S.S.M., Scharmga A.M.J., Schols R.M.M., van Weezelenburg M.A.S., Cau R., van der Hulst R.R.W.J.M., MicroSurgical Robot Research Group (2022). One-Year Outcomes of the First Human Trial on Robot-Assisted Lymphaticovenous Anastomosis for Breast Cancer–Related Lymphedema. Plast. Reconstr. Surg..

[59] O’connor E.F., Rozen W.M., Chowdhry M., Band B., Ramakrishnan V.V., Griffiths M. (2016). Preoperative computed tomography angiography for planning DIEP flap breast reconstruction reduces operative time and overall complications. Gland. Surg..

[60] Broer P.N., Weichman K.E., Tanna N., Wilson S., Ng R., Ahn C., Choi M., Karp N.S., Levine J.P., Allen R.J. (2013). Venous coupler size in autologous breast reconstruction—does it matter?. Microsurgery.

[61] Rogoń I., Rogoń A., Kaczmarek M., Bujnowski A., Wtorek J., Lachowski F., Jankau J. (2024). Flap Monitoring Techniques: A Review. J. Clin. Med..

[62] Hölzle F., Loeffelbein D.J., Nolte D., Wolff K.-D. (2006). Free flap monitoring using simultaneous non-invasive laser Doppler flowmetry and tissue spectrophotometry. J. Cranio-Maxillofac. Surg..

[63] Keller A. (2007). Noninvasive Tissue Oximetry for Flap Monitoring: An Initial Study. J. Reconstr. Microsurg..

[64] Keller A. (2009). A New Diagnostic Algorithm for Early Prediction of Vascular Compromise in 208 Microsurgical Flaps Using Tissue Oxygen Saturation Measurements. Ann. Plast. Surg..

[65] Schoenbrunner A., Hackenberger P.N., DeSanto M.B., Chetta M. (2021). Cost-Effectiveness of Vioptix versus Clinical Examination for Flap Monitoring of Autologous Free Tissue Breast Reconstruction. Plast. Reconstr. Surg..

[66] Mericli A.F., Wren J., Garvey P.B., Liu J., Butler C.E., Selber J.C. (2017). A Prospective Clinical Trial Comparing Visible Light Spectroscopy to Handheld Doppler for Postoperative Free Tissue Transfer Monitoring. Plast. Reconstr. Surg..

[67] Smit J., Whitaker I., Liss A., Audolfsson T., Kildal M., Acosta R. (2009). Post operative monitoring of microvascular breast reconstructions using the implantable Cook–Swartz doppler system: A study of 145 probes & technical discussion. J. Plast. Reconstr. Aesthetic Surg..

[68] Um G.T., Chang J., Louie O., Colohan S.M., Said H.K., Neligan P.C., Mathes D.W. (2014). Implantable Cook-Swartz Doppler probe versus Synovis Flow Coupler for the post-operative monitoring of free flap breast reconstruction. J. Plast. Reconstr. Aesthetic Surg..

[69] Rozen W.M.M., Whitaker I.S.M., Wagstaff M.J.D.M., Audolfsson T., Acosta R. (2010). Buried Free Flaps for Breast Reconstruction: A New Technique Using the Cook-Swartz Implantable Doppler Probe for Postoperative Monitoring. Plast. Reconstr. Surg..

[70] Frost M.W., Niumsawatt V., Rozen W.M., Eschen G.E.T., Damsgaard T.E., Kiil B.J. (2015). Direct comparison of postoperative monitoring of free flaps with microdialysis, implantable cook-swartz Doppler probe, and clinical monitoring in 20 consecutive patients. Microsurgery.

[71] A Agha R., Gundogan B., Fowler A.J., Bragg T.W.H., Orgill D.P. (2014). The efficacy of the Cook-Swartz implantable Doppler in the detection of free-flap compromise: A systematic review protocol. BMJ Open.

[72] Chadwick S., Khaw R., Duncan J., Wilson S., Highton L., O’CEallaigh S. (2020). The use of venous anastomotic flow couplers to monitor buried free DIEP flap reconstructions following nipple-sparing mastectomy. JPRAS Open.

[73] Udesen A., Løntoft E., Kristensen S.R. (2000). Monitoring of Free Tram Flaps with Microdialysis. J. Reconstr. Microsurg..

[74] Whitaker I., Rozen W., Chubb D., Acosta R., Kiil B., Birke-Sorensen H., Grinsell D., Ashton M. (2010). Postoperative Monitoring of Free Flaps in Autologous Breast Reconstruction: A Multicenter Comparison of 398 Flaps Using Clinical Monitoring, Microdialysis, and the Implantable Doppler Probe. J. Reconstr. Microsurg..

[75] Hirigoyen M.B., Blackwell K.E., Zhang W.X., Silver L., Weinberg H., Urken M.L. (1997). Continuous Tissue Oxygen Tension Measurement as a Monitor of Free-Flap Viability. Plast. Reconstr. Surg..

[76] Arnež Z.M., Ramella V., Papa G., Novati F.C., Manca E., Leuzzi S., Stocco C. (2019). Is the LICOX® PtO_2_ system reliable for monitoring of free flaps? Comparison between two cohorts of patients. Microsurgery.

[77] Shadid O., Seth I., Cuomo R., Rozen W.M., Marcaccini G. (2025). Artificial Intelligence in Microsurgical Planning: A Five-Year Leap in Clinical Translation. J. Clin. Med..

[78] Allen B., University Of Nevada Las (2025). The Present and Future of Robotic Surgery in Breast Cancer and Breast Reconstruction. Clin. Stud. Med Case Rep..

